# Safety Evaluation of *Bacillus* Isolates From Fermented Tarhana With Probiotic Potential

**DOI:** 10.1002/fsn3.72372

**Published:** 2026-09-24

**Authors:** Ayşe Avcı, Esma Alemdar, Elif Sezer, İnci Cerit, Fikriye Alev Akçay, İbrahim Çakır

**Affiliations:** ^1^ Department of Food Engineering Sakarya University Sakarya Türkiye; ^2^ Department of Engineering Bolu Abant Izzet Baysal University Bolu Türkiye

**Keywords:** *Bacillus*, bile resistance, probiotics, tarhana

## Abstract

Tarhana is a Turkish fermented food that has been widely consumed by Turkish people for hundreds of years. The main fermentation microorganisms are lactic acid bacteria; however, in this study, we aimed to isolate *Bacillus* strains from tarhana samples and to investigate some probiotic properties after identification. Various types of tarhana samples were collected, and after a preliminary screening test, 53 isolates were selected. The isolates were Gram‐positive, endospore‐forming, catalase‐positive, and motile. Biochemical pathogenicity tests, including lecithinase, DNase, and hemolytic activities, were performed, and all isolates were lecithinase‐negative and DNase‐negative. Hemolytic tests indicated that eight isolates were gamma‐hemolytic and the rest were alpha‐hemolytic. Ten virulence genes were investigated in all isolates, and none of them harbored any of these genes. The isolates exhibited strong tolerance to bile salts (78.3%–97.3%) and simulated gastric conditions (81.3%–99.5%). The hydrophobicity potential of the isolates was quite variable, ranging from 14.6% to 76.2%. Aggregation after 24 h ranged from 50.4% to 79.2%. Twenty of the isolates were strong biofilm formers and were susceptible to the antibiotics specific to *Bacillus* (according to EUCAST). This study revealed the potential of Turkish tarhana as a source of probiotic *Bacillus* strains.

## Graphical abstract

Fifty‐three *Bacillus* strains were isolated from traditional tarhana. MALDI‐TOF MS identified the isolates within the *Bacillus subtilis* group. None of the isolates harbored the tested virulence genes. The isolates exhibited high tolerance to bile salts and simulated gastric conditions. Several isolates exhibited excellent probiotic characteristics.

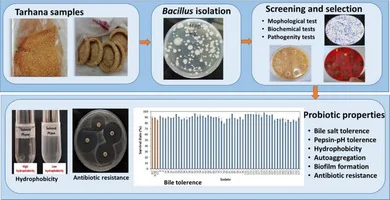

## 1 Introduction

The intestinal microbiota plays a crucial role in maintaining human and animal health through its involvement in nutrition, immune responses, and physiological functions. Under normal conditions, beneficial and harmful microorganisms in the gastrointestinal tract exist in a balanced state; however, this balance can be disrupted by disease, stress, irregular dietary habits, environmental factors, and antibiotic use, which may also reduce beneficial bacterial populations. Probiotic microorganisms have been shown to play an important role in restoring and regulating the intestinal microbiota (Isolauri et al. 2004; Lee et al. 2019; Salminen et al. 1999). The term probiotic is derived from the Greek words pro and bios, meaning “for life,” (Soccol et al. 2010) and is defined by the FAO and the WHO as live microorganisms that, when administered in adequate amounts, confer a health benefit on the host (FAO/WHO 2002). Probiotics can be utilized through foods or directly and have been reported to shorten infection duration and/or reduce susceptibility to pathogens in humans and animals (Sorokulova et al. 2008). In addition, they contribute to host health by strengthening the intestinal barrier and immune system, preventing epithelial damage, and exhibiting antagonistic or antimicrobial activity against enteropathogenic bacteria through bacteriocin production or metabolic interactions (Lee et al. 2019; Zulkhairi Amin et al. 2019).

Probiotic organisms must tolerate harsh gastric conditions during transit through the gastrointestinal tract and demonstrate adhesion capability to human epithelial cells to exert beneficial effects (Ahire et al. 2020). Lactic acid bacteria (LAB) and *Bifidobacterium* spp. are globally acknowledged as the predominant probiotic bacterial groups owing to their documented health benefits. Moreover, specific strains belonging to *Enterococcus*, *Streptococcus*, and *Bacillus* spp., together with certain *Saccharomyces* spp., have also been incorporated into probiotic formulations (Elshaghabee et al. 2017). In particular, *Bacillus* spp. exhibit notable advantages as probiotic candidates owing to their sporulation characteristics. Spore formation provides effective protection against hostile gastrointestinal conditions, including low pH, bile salts, and digestive enzymes as well as enhanced resistance to harsh industrial processing and storage (Shinde et al. 2019).

Tarhana is a traditional fermented food of Turkish cuisine, produced by fermenting a dough composed mainly of wheat flour and yogurt for a duration of 1–7 days. Baker's yeast is commonly added during dough preparation to support the fermentation process. Following fermentation, the product is dried under open‐air conditions and consumed as a soup, owing to its high nutritional value and richness in proteins and vitamins. Various ingredients, including salt, red pepper, tomato, and onion, are incorporated to enhance the sensory properties of the final product (Avcı et al. 2019; Ibanoglu et al. 1995). Tarhana exhibits considerable regional variability in its production methods, and the Uşak and Maraş varieties are protected by geographical indication status in Türkiye (Çekal and Aslan 2017). The fermentation process is primarily driven by yeasts and LAB. Metabolic compounds produced by LAB play a key role in the development of tarhana's characteristic flavor and aroma, while simultaneously enhancing product stability by increasing the acidity of the fermentation environment (Şimşek et al. 2017). In addition to LAB and *Saccharomyces cerevisiae* , tarhana has been reported to harbor *Bacillus* species adapted to food‐related environments (Avcı et al. 2021). Therefore, the rich microbial diversity of traditional tarhana represents an underexplored reservoir of potentially beneficial microorganisms. Despite the widespread consumption of tarhana, scientific evidence regarding the probiotic potential of spore‐forming *Bacillus* species isolated from this traditional fermented food remains limited. Accordingly, the aim of this study was to conduct a comprehensive screening of *Bacillus* isolates obtained from traditional Turkish fermented cereal‐based foods to identify promising food‐grade probiotic candidates. Essential probiotic properties, including bile tolerance and simulated gastric tolerance, hydrophobicity, and auto‐aggregation, were evaluated.

## 2 Materials and Methods

### 2.1 Materials

Unless otherwise stated, all culture media, chemicals, molecular biology reagents, and antibiotic discs used in this study were of analytical grade and purchased from commercial suppliers. Egg yolk agar Base, DNase agar, and pepsin (1:1000) were obtained from HiMedia Laboratories (Mumbai, India). Nutrient broth and boric acid were purchased from Merck (Darmstadt, Germany), whereas bacteriological agar and DNase agar were obtained from Condalab (Madrid, Spain). PCR primers were synthesized by Oligomer Biotechnology (Ankara, Türkiye). MgCl_2_ and PCR‐grade water were purchased from Ampliqon (Odense, Denmark), whereas *Taq* DNA polymerase and 6× DNA loading dye were obtained from Thermo Fisher Scientific (Waltham, MA, USA). Tris base was purchased from Sigma‐Aldrich (St. Louis, MO, USA), EDTA from Thermo Scientific (Rockford, IL, USA), agarose from PRONA BioMax (Madrid, Spain), and DNA‐Dye NonTox from AppliChem GmbH (Darmstadt, Germany). Antibiotic discs were obtained from Bioanalyse (Ankara, Türkiye). Bile salts and n‐hexadecane were purchased from Honeywell Fluka (Charlotte, NC, USA) and TCI Chemicals (Tokyo, Japan), respectively.

### 2.2 Isolation and Preliminary Screening of *Bacillus* Isolates

Eighty‐three homemade tarhana samples were collected from 13 cities in Türkiye. The samples were collected in plastic bags and stored at room temperature. For isolation, 5 g of each tarhana sample was homogenized in 45 mL of physiological saline solution (0.85% NaCl). The suspensions were heated at 80°C for 30 min to inhibit non‐spore‐forming bacteria, followed by serial dilution (10^−2^–10^−5^), and the resulting dilutions were spread onto nutrient agar plates. The plates were incubated at 37°C for 24 h, after which colonies exhibiting distinct morphological features were selected. The purity of the cultures was confirmed through three repeated streakings on nutrient agar (Akçay 2017). For long‐term storage, the isolates were preserved in nutrient broth supplemented with 25% (v/v) glycerol at −45°C. A total of 466 bacteria were isolated and subjected to preliminary identification tests, including Gram and spore staining, colony and cell morphologies, catalase test, and motility. In addition, pathogenicity‐related assays such as lecithinase, DNase, and hemolytic activities were performed. Based on the results of the preliminary evaluations, 53 non‐pathogenic and spore‐forming isolates with distinct morphological characteristics were selected for subsequent probiotic characterization, the results of which are presented in this study. Table 1 presents the regions of the samples collected, types of tarhana samples, and isolate codes. Three commercial probiotic strains, *Bacillus clausii* MTCC 5472 (CL), *Bacillus coagulans* MTCC 5856 (BCO), and *B. clausii* strains O/C, N/R, SIN, and T (Enterogermina; BCE), were included as reference strains throughout the study.

**TABLE 1:** The source and properties of the tarhana samples used for *Bacillus* isolation.

The region of the tarhana sample	Code of the samples collected	Type of tarhana	Number of isolates per sample	Code of the isolates
Kocaeli	KSGA	Dried powder	2	8, 9
	KSGB	Dried powder	1	10
Tokat	TAK	Dried wheat	2	14, 16
	TAR	Dried wheat	1	21
	TTA	Dried wheat	1	25
Sakarya	SCT	Dried powder	2	28, 29
	SMK	Dried powder	1	34
	SMM	Dried powder	2	37, 38
	SPKA	Dried powder	1	45
	SST	Dried powder	2	50, 52
	SSTY	Wet dough	1	54
Tekirdağ	TEB	Dried powder	1	61
	TEC	Dried powder	1	63
	TEM	Dried powder	2	66, 67
	TET	Dried powder	2	69, 70
Kahramanmaraş	MAC	Dried pellets	3	76, 77, 79
	MAD	Dried pellets	2	80, 81
	MAE	Dried pellets	3	85, 87, 88
	MAF	Dried pellets	1	91
Gaziantep	GCB	Dried wheat	2	96, 101
	GCD	Dried wheat	1	107
Uşak	UCK	Dried powder	3	113, 115, 116
	UGK	Dried powder	2	117, 119
	UHC	Dried powder	1	121
	UFA	Dried powder	1	123
	USD	Dried powder	1	124
	USK	Dried powder	1	127
Çankırı	CEF	Dried powder	1	134
	CEM	Dried powder	1	137
	CFC	Dried powder	3	139, 140, 142
	CHA	Dried powder	1	143
	CHT	Dried powder	1	146
Bolu	BHK	Cornelian cherry	1	155
	BKT	Cornelian cherry	1	156
Erzincan	EHT	Dried wheat	1	157

### 2.3 Morphological and Biochemical Characterization of Isolates

The isolates were initially characterized based on colony morphology, Gram reaction, endospore formation, catalase activity, and motility. Colony morphology was evaluated on nutrient agar plates, whereas Gram and spore staining were performed to determine the Gram reaction and endospore formation, respectively. Catalase activity was assessed by adding 3% hydrogen peroxide to fresh cultures, and motility was determined using semi‐solid nutrient agar (0.5% agar) (Cappucino and Welsh 2018).

### 2.4 MALDI‐TOF MS Identification of the Isolates

Presumptive species identification of the isolates was performed using matrix‐assisted laser desorption/ionization time‐of‐flight mass spectrometry (MALDI‐TOF MS) with an Autoflex Speed instrument (Bruker Daltonics GmbH, Bremen, Germany). Prior to MALDI‐TOF MS analysis, isolates were cultured on nutrient agar at 25°C for 18 h. A fresh colony from each isolate was directly transferred onto an MTP 384 Ground Steel Target plate and overlaid with α‐cyano‐4‐hydroxycinnamic acid (HCCA, 10 mg/mL) matrix solution. The instrument was calibrated using the Bruker Bacterial Test Standard (BTS). Mass spectra were processed using Biotyper software version 3.4 (Söylemez‐Milli et al. 2025). Identification scores were interpreted according to the manufacturer's recommended criteria: scores ≥ 2.000 were considered reliable for species‐level identification, scores between 1.700 and 1.999 indicated reliable genus‐level identification with probable species assignment, and scores < 1.700 were considered unreliable (Schulthess et al. 2014).

### 2.5 Safety Characterization of the Isolates

#### 2.5.1 Lecithinase Activity

Lecithinase activity was determined using egg yolk agar base supplemented with 10% (v/v) egg yolk under aseptic conditions before pouring the medium into Petri plates. Following inoculation of the isolates onto the medium, the plates were incubated at 37°C for 24 h. The isolates that produced an opaque, yellowish precipitation zone around the colonies were considered lecithinase‐positive (Fritze and Pukall 2001).

#### 2.5.2 Hemolytic Activity

For the determination of hemolytic activity, all isolates were streaked onto sheep blood agar plates and incubated at 37°C for 24 h. After incubation, the hemolysis patterns were evaluated as follows: greenish discoloration around the colonies was interpreted as α‐hemolysis, clear zones as β‐hemolysis, and the absence of any zone as γ‐hemolysis (Ritter et al. 2018).

#### 2.5.3 DNase Activity

DNase activity was determined by streaking the isolates onto DNase agar and incubating them at 37°C for 24 h. After incubation, the plates were flooded with 1 N HCl, and the formation of a clear halo zone around the growth was considered a positive DNase reaction (Taheur et al. 2016).

#### 2.5.4 Determination of Virulence Genes

The presence of toxin‐associated genes, including the hemolysin BL complex (*hblA*, *hblC*, *hblD*), non‐hemolytic enterotoxin complex (*nheA, nheB, nheC*), enterotoxin T (*bceT*), enterotoxin FM (*entFM*), cytotoxin K (cytK), and the emetic toxin cereulide synthetase genes (*cesB*), was investigated by using polymerase chain reaction (PCR) (Celandroni et al. 2019; Bianco et al. 2023). Genomic DNA was extracted using the GeneMATRIX Tissue & Bacterial DNA Purification Kit (E3551‐0S; EURx, Gdańsk, Poland) according to the manufacturer's instructions. DNA concentration and purity were determined by measuring the absorbance at 260 and 280 nm using a Multiskan SkyHigh microplate spectrophotometer (Thermo Fisher Scientific, Waltham, MA, USA) equipped with a μDrop Duo Plate. Primers targeting these genes were designed by a national reference laboratory (Oligomer Biotechnology; Ankara, Türkiye). PCRs were carried out by using a Bio‐Rad T100 thermal cycler (Bio‐Rad, Hercules, CA, USA). Initially, a gradient PCR was performed to determine the optimal annealing temperatures for each primer pair. For this purpose, three *Bacillus cereus* strains (SBT8, EKT1, and KET2) were used as controls, and annealing temperatures ranging from 45°C to 55°C were tested. The primer‐specific annealing temperatures yielding the best amplification efficiency were selected and subsequently used for PCR amplification of all isolates. The primer sequences and optimized annealing temperatures are shown in Table 2 (Jeon et al. 2018). The magnesium chloride concentration was also optimized by testing 1.5, 2, 4, and 6 mM MgCl₂, with 4 mM yielding the best amplification results. Template DNA concentrations were adjusted to 25–50 ng/μL. Each PCR reaction contained 0.24 mM dNTP mix, 0.3 μL of each primer (100 μM stock), 0.2 μL Taq DNA polymerase (5 U/μL), and nuclease‐free water, for a final volume of 25 μL. The PCR conditions were as follows: initial denaturation at 94°C for 3 min; denaturation at 94°C for 30 s; annealing at the primer‐specific (optimized gradient value) for 30 s; extension at 72°C for 1 min; number of cycles: 34; final extension at 72°C for 5 min. After the amplification, agarose gel electrophoresis was performed using a PowerPac Basic Power Supply (Bio‐Rad Laboratories, Hercules, CA, USA) to analyze the PCR products at 1.25% agarose in 1× Tris‐boric acid‐EDTA buffer (TBE buffer). DNA samples were stained with DNA‐Dye NonTox (A9555; AppliChem GmbH, Darmstadt, Germany). The electrophoresis was carried out at 120–150 V for approximately 30 min, and the DNA bands were subsequently visualized using a TCP‐26.MC UV transilluminator (Vilber Lourmat, Collégien, France).

**TABLE 2:** Primers used for the determination of virulence genes.

Primers	Primer sequence (5′ → 3′)	Size	Annealing temperature (°C)
		(base pairs, bp)	
*hblA‐F*	AAGCAATGGAATACAATGGG	1154	50
*hblA‐R*	AGAATCTAAATCATGCCACTGC		
*hblC‐F*	GATACTCAATGTGGCAACTGC	740	47
*hblC‐R*	TTGAGACTGCTCGTCTAGTTG		
*hblD‐F*	ACCGGTAACACTATTCATGC	829	50
*hblD‐R*	GAGTCCATATGCTTAGATGC		
*nheA‐F*	GTTAGGATCACAATCACCGC	755	50
*nheA‐R*	ACGAATGTAATTTGAGTCGC		
*nheB‐F*	TTTAGTAGTGGATCTGTAGC	743	48
*nheB‐R*	TTAATGTTCGTTAATCCTGC		
*nheC‐F*	TGGATTCCAAGATGTAACG	683	49
*nheC‐R*	ATTACGACTTCTGCTTGTGC		
*ces‐F*	GGTGACACATTATCATATAAGGTG	1271	55
*ces‐R*	GTAAGCGAACCTGTCTGTAACAACA		
*bcet‐F*	CGTATCGGTCGTTCACTCGG	661	55
*bcet‐R*	GTTGATTTTCCGTAGCCTGGG		
*entFM‐F*	ATGAAAAAAGTAATTTGCAGG	1269	50
*entFM‐R*	TTAGTATGCTTTTGTGTAACC		
*cytK‐F*	GTAACTTTCATTGATGATCC GAATACATAAATAATTGGTTTCC	505	47
*cytK‐R*			

#### 2.5.5 Antibiotic Susceptibility Tests

Antibiotic susceptibility testing of the isolates was performed using antibiotics recommended by the European Committee on Antimicrobial Susceptibility Testing (EUCAST; www.eucast.org) for *Bacillus* species. The selection of antibiotics and interpretation of the results were based on the EUCAST Clinical Breakpoint Tables v.15.0 (EUCAST 2025). The following antibiotic disks were used at the concentrations recommended by EUCAST: imipenem (IPM10; 10 μg), meropenem (MEM10; 10 μg), ciprofloxacin (CIP5; 5 μg), levofloxacin (LEV5; 5 μg), vancomycin (VA5; 5 μg), erythromycin (E15; 15 μg), clindamycin (DA2; 2 μg), and linezolid (LNZ10; 10 μg). Mueller–Hinton agar was used as the test medium, and the plates were incubated at 35°C for 18 h, following EUCAST guidance. After incubation, inhibition zone diameters were measured and evaluated according to the EUCAST 2025 criteria, classifying isolates as susceptible (S), Susceptible, increased exposure (I), or resistant (R).

### 2.6 Evaluation of Probiotic Properties

#### 2.6.1 Bile Salt Tolerance

For the bile salt tolerance assay, 2 mL of actively growing cultures (10^9^ CFU/mL) were inoculated into 20 mL of fresh nutrient broth supplemented with 0.3% bile salts and incubated at 37°C for 3 h under shaking conditions (120 rpm) using an Incu‐Shaker Mini (Benchmark Scientific, Sayreville, NJ, USA). Samples were collected at 0 and 3 h, serial dilutions were prepared, and plated on nutrient agar. The logarithmic change in bacterial counts was used to express resistance as the percentage of viable cells remaining (Equation 1), that is, the survival rate (Lee et al. 2017).

(1) $$\text{Survival rate}\;\left(\%\right)=\frac{\left(\log N_{0}-\log N_{t}\right)}{\log N_{0}}\times 100$$

where *N*_0_ and *N*_*t*_ are the number of cells at time zero and after 3 h of incubation with bile salts, respectively.

#### 2.6.2 Resistance to Simulated Gastric Conditions

To determine the resistance to simulated gastric juice, 2 mL of actively growing vegetative cells (10^9^ CFU/mL) were inoculated into a nutrient broth medium containing 0.3% pepsin and the pH was adjusted to 2.5 using 0.1 M HCl. The cultures were incubated at 37°C under shaking conditions (120 rpm) for 3 h. Serial dilutions were prepared from the samples collected at the beginning and the end of the incubation, and serially diluted samples were plated onto nutrient agar to determine viable cell counts (Lee et al. 2017). The percentage of resistance to simulated gastric juice was calculated based on the logarithmic reduction in cell numbers (Equation 1).

#### 2.6.3 Hydrophobicity

Isolates grown in nutrient broth at 37°C and 120 rpm for 24 h were harvested by centrifugation (9000× *g*, 10 min). The cell pellets were washed thrice with phosphate‐buffered saline (PBS, pH 7.2) by resuspension and centrifugation under the same conditions. The optical densities (OD_600_) were adjusted to 0.5–0.6 using a UV–VIS spectrophotometer (Shimadzu Mini UV‐1240, Kyoto, Japan). Cell suspensions were mixed by vortexing with equal volumes of n‐hexadecane for 60 s. Then, the samples were allowed to stand at room temperature for 1 h without agitation, and the separated aqueous phase was collected to measure the absorbance at 600 nm using a UV–VIS spectrophotometer. The percentage of hydrophobicity was calculated using (Equation 2) (Shivangi et al. 2020).

(2) $$\text{Hidrophobicity}\;\left(\%\right)=\frac{A_{0}-A_{t}}{A_{0}}\times 100$$

where *A*_0_ is the absorbance value of the culture before the addition of n‐hexadecane, and *A*_*t*_ is the absorbance value of the aqueous phase after 1 h.

#### 2.6.4 Auto‐Aggregation

To evaluate auto‐aggregation, 4 mL of the cell suspension (OD_600_: 0.5–0.6) prepared as described above was vortexed for 10 s and then incubated at 37°C for 24 h without agitation. The upper phase (1 mL) was collected at 0, 3, and 24 h, and the optical density (OD) was measured at 600 nm using a UV–VIS spectrophotometer. Auto‐aggregation was calculated using the (Equation 3) (Angmo et al. 2016).

(3) $$\text{Auto}-\text{aggregation}\;\left(\%\right)=\left[1-A_{t}/A_{0}\right]\times 100$$

*A*_*t*_ and *A*_0_ were the absorbance values measured at time *t* and at the beginning, respectively.

#### 2.6.5 Biofilm Formation

Biofilm formation was determined using the crystal violet assay with 96‐well microtiter plates (Sudan and Li 2022). Following overnight incubation of the isolates in nutrient broth at 37°C, 15 μL of each culture (10^9^ CFU/mL) was inoculated into wells containing 150 μL of nutrient broth. The microplates were then incubated statically at 37°C for 24–72 h. After incubation, the liquid phase was removed, and the wells were gently washed twice with 165 μL of PBS. The plates were air‐dried at room temperature, and 165 μL of 0.1% crystal violet solution was added to each well and allowed to stain the biofilm for 30 min at room temperature. Following staining, the dye solution was discarded, and the wells were washed twice with PBS. The stained wells were air‐dried at room temperature, and 165 μL of 70% ethanol was added to solubilize the bound dye. After 30 min, the contents were transferred to a new microplate, and absorbance values were measured at 595 nm using a microplate reader (Thermo Scientific, Multiskan SkyHigh, Waltham, MA, USA). Biofilm production performance was classified based on absorbance values as follows: abs < 0.124: weak; 0.124–0.240: moderate; > 0.240: strong (Mathur et al. 2006).

### 2.7 Statistical Analysis

All experiments were performed in triplicate, and the results are presented as mean ± standard deviation (SD). Descriptive statistical analyses were performed to summarize the experimental data using IBM SPSS Statistics software (version 20, IBM Corp., Armonk, NY, USA).

## 3 Results and Discussion

### 3.1 Morphological and Biochemical Characterization

Based on the results of the preliminary characterization and safety screening, 53 isolates were selected for further analyses. All selected isolates were Gram‐positive, spore‐forming, catalase‐positive, motile, rod‐shaped bacteria with typical *Bacillus* cell morphology (Table 3).

**TABLE 3:** Presumptive species identification by MALDI‐TOF MS, phenotypic characteristics, and safety‐related traits of *Bacillus* isolates.

Isolate No.	Presumptive species‐level identification by MALDI‐TOFMS	MALDI‐TOF MS score	Gram reaction	Endospore formation	Catalase activity	Motility	Lecithinase activity	DNase activity	Hemolytic activity	Presence of virulence genes*
8	NI		+	+	+	+	−	−	*α*	ND
9	NI		+	+	+	+	−	−	*α*	ND
10	*B. mojavensis*	1.705	+	+	+	+	−	−	*α*	ND
14	*B. mojavensis*	2.087	+	+	+	+	−	−	*α*	ND
16	NI		+	+	+	+	−	−	*α*	ND
21	*B. mojavensis*	1.891	+	+	+	+	−	−	*α*	ND
25	*B. mojavensis*	2.115	+	+	+	+	−	−	*γ*	ND
28	*B. subtilis*	1.928	+	+	+	+	−	−	*α*	ND
29	*B. subtilis*	1.710	+	+	+	+	−	−	*α*	ND
34	*B. mojavensis*	1.742	+	+	+	+	−	−	*γ*	ND
37	*B. subtilis*	2.067	+	+	+	+	−	−	*α*	ND
38	*B. mojavensis*	1.993	+	+	+	+	−	−	*α*	ND
45	*B. mojavensis*	1.950	+	+	+	+	−	−	*α*	ND
50	*B. mojavensis*	1.995	+	+	+	+	−	−	*α*	ND
52	*B. vallismortis*	2.051	+	+	+	+	−	−	*γ*	ND
54	*B. mojavensis*	2.099	+	+	+	+	−	−	*α*	ND
61	*B. vallismortis*	1.734	+	+	+	+	−	−	*α*	ND
63	*B. vallismortis*	1.773	+	+	+	+	−	−	*α*	ND
66	*B. subtilis*	1.993	+	+	+	+	−	−	*α*	ND
67	*B. vallismortis*	1.765	+	+	+	+	−	−	*α*	ND
69	*B. vallismortis*	1.851	+	+	+	+	−	−	*α*	ND
70	*B. vallismortis*	1.879	+	+	+	+	−	−	*α*	ND
76	*B. subtilis*	1.729	+	+	+	+	−	−	*α*	ND
77	*B. vallismortis*	1.825	+	+	+	+	−	−	*α*	ND
79	*B. subtilis*	1.746	+	+	+	+	−	−	*γ*	ND
80	*B. vallismortis*	1.983	+	+	+	+	−	−	*α*	ND
81	*B. subtilis*	1.841	+	+	+	+	−	−	*α*	ND
85	*B. vallismortis*	1.700	+	+	+	+	−	−	*α*	ND
87	*B. subtilis*	1.768	+	+	+	+	−	−	*α*	ND
88	*B. mojavensis*	2.014	+	+	+	+	−	−	*α*	ND
91	*B. vallismortis*	1.987	+	+	+	+	−	−	*α*	ND
96	*B. vallismortis*	1.863	+	+	+	+	−	−	*α*	ND
101	*B. vallismortis*	1.986	+	+	+	+	−	−	*α*	ND
107	*B. subtilis*	1.759	+	+	+	+	−	−	*γ*	ND
113	NI		+	+	+	+	−	−	*α*	ND
115	*B. subtilis*	2.218	+	+	+	+	−	−	*α*	ND
116	*B. subtilis*	2.000	+	+	+	+	−	−	*γ*	ND
117	*B. vallismortis*	1.933	+	+	+	+	−	−	*γ*	ND
119	*B. vallismortis*	2.010	+	+	+	+	−	−	*α*	ND
121	*B. vallismortis*	1.947	+	+	+	+	−	−	*α*	ND
123	*B. vallismortis*	1.857	+	+	+	+	−	−	*α*	ND
124	*B. vallismortis*	1.927	+	+	+	+	−	−	*α*	ND
127	*B. vallismortis*	1.954	+	+	+	+	−	−	*α*	ND
134	*B. subtilis*	2.006	+	+	+	+	−	−	*α*	ND
137	*B. vallismortis*	2.084	+	+	+	+	−	−	*α*	ND
139	*B. mojavensis*	1.877	+	+	+	+	−	−	*α*	ND
140	*B. vallismortis*	1.735	+	+	+	+	−	−	*α*	ND
142	*B. mojavensis*	1.876	+	+	+	+	−	−	*α*	ND
143	*B. mojavensis*	1.949	+	+	+	+	−	−	*α*	ND
146	*B. mojavensis*	1.930	+	+	+	+	−	−	*α*	ND
155	*B. mojavensis*	2.198	+	+	+	+	−	−	*α*	ND
156	*B. vallismortis*	1.735	+	+	+	+	−	−	*α*	ND
157	*B. vallismortis*	1.982	+	+	+	+	−	−	*γ*	ND

Abbreviations: +, positive reaction; −, negative reaction; *α*, alpha hemolytic; *γ*, gamma hemolytic; ND, Not detected; NI, not reliable identification.

* Virulence genes investigated: *nheA*, *nheB*, *nheC*, *hblA*, *hblC*, *hblD*, *cytK*, *ces, bcet, and entFM*.

### 3.2 MALDI‐TOF MS Identification

A total of 53 isolates were analyzed by MALDI‐TOF MS for presumptive species identification. Reliable identification was achieved for 49 (92.5%) isolates, whereas four isolates (7.5%) could not be reliably identified (NI). MALDI‐TOF MS identification scores ranged from 1.700 to 2.218. Among the identified isolates, *Bacillus vallismortis* was the predominant species, accounting for 22 isolates (44.9%), followed by *Bacillus mojavensis* with 15 isolates (30.6%) and *Bacillus subtilis* with 12 isolates (24.5%). All the identified isolates belonged to the *B. subtilis* group (Xu and Kovács 2024). The occurrence of *B. vallismortis* , *B. mojavensis* , and *B. subtilis* in homemade tarhana is consistent with previous reports describing members of the *B. subtilis* species group in a variety of traditional fermented foods (Park et al. 2010; Li et al. 2022). Although only 13 isolates achieved MALDI‐TOF MS scores ≥ 2.000, all remaining identified isolates yielded scores between 1.700 and 1.999, corresponding to reliable genus‐level identification with probable species assignment according to the manufacturer's recommended criteria (Table 3). Previous studies have shown that the quality of MALDI‐TOF MS spectra obtained from members of the *Bacillus* genus may be influenced by culture conditions, including incubation temperature, incubation time, and endospore formation. Fresh vegetative cells generally produce higher‐quality spectra, thereby improving identification accuracy (Shu and Yang 2017). Accordingly, the isolates in the present study were cultured on nutrient agar at 25°C for 18 h prior to MALDI‐TOF MS analysis. Despite these optimized conditions, four isolates could not be reliably identified, most likely because their highly mucoid phenotype, associated with excessive exopolysaccharide production, interfered with the acquisition of high‐quality mass spectra.

### 3.3 Safety Characterization

#### 3.3.1 Lecithinase Activity

Lecithinase activity was performed on egg yolk agar plates and the results indicated that the isolates did not have lecithinase activity (Table 3). Presence of this enzyme is considered an indicator of potential pathogenicity and the members of the *Bacillus cereus* group (*B. cereus, B. anthracis, B. pyogenes, B. mycoides, B. thuringiensis*) are known to exhibit lecithinase activity (Hwang and Park 2015). Therefore, the isolates were considered safe in this regard. Lecithinase hydrolyzes lecithin contained in egg yolk agar, resulting in the formation of an opaque precipitation zone surrounding the colonies. The enzyme belongs to the phospholipase family (phosphatidylcholine hydrolase and phospholipase C) and acts by cleaving the ester bonds of lecithin. Hydrolysis of lecithin, an essential component of cell membranes, leads to the production of diglycerides and phosphorylcholine. The breakdown of membrane phospholipids ultimately results in cell lysis (Schraft and Griffiths 1995; Sharaf et al. 2014).

#### 3.3.2 DNase Activity

Deoxyribonuclease (DNase) production is considered an important virulence‐associated characteristic in many pathogenic bacteria, as extracellular deoxyribonucleases degrade extracellular DNA and contribute to bacterial pathogenicity (Borthakur et al. 2025). Therefore, DNase activity was evaluated in all isolates as part of the safety assessment. None of the isolates exhibited DNase activity, supporting their safety profile as potential probiotic candidates (Table 3).

#### 3.3.3 Hemolytic Activity

Hemolysis refers to the lysis of red blood cells and the subsequent release of hemoglobin. The presence of hemolytic activity is considered undesirable for probiotic strains, as it may indicate potential pathogenicity. Using sheep blood agar, hemolysis can be categorized into three distinct types. The first type, β‐hemolysis, results from the complete hydrolysis of red blood cells and appears as a clear, transparent zone surrounding the colonies. The second type, α‐hemolysis, is characterized by a greenish‐brown discoloration around the colonies due to partial hemoglobin degradation, leading to accumulation of the intermediate product methemoglobin. The third type, γ‐hemolysis, indicates the absence of hemolytic activity, in which no visible zone or discoloration is observed around colony growth (Kim et al. 2022). Among the 53 isolates tested, none were β‐hemolytic, eight isolates (isolates #25, 34, 52, 79,107, 116, 117, and 157) were γ‐hemolytic, and the others were α‐hemolytic (Table 3). Although hemolysin production is generally considered a virulence‐associated trait, hemolytic activity in *Bacillus* species may also result from the action of fibrinolytic enzymes and various lipopeptides, including surfactin, fengycin, and iturin. Therefore, the assessment of hemolysis in *Bacillus* strains should be interpreted in conjunction with additional enzymatic and safety‐related parameters rather than being evaluated in isolation. Moreover, hemolytic effects are thought to be relevant primarily when bacteria translocate into the bloodstream through a compromised intestinal barrier; however, in vivo studies have demonstrated that orally administered *B. subtilis* spores do not cross mucosal surfaces (Ruiz Sella et al. 2021). Similarly, Zulkhairi Amin et al. (2019) reported that *B. amyloliquefaciens* HTI‐19 displayed α‐hemolytic, while *B. subtilis* HTI‐23 was γ‐hemolytic, emphasizing that only β‐hemolysis should be considered detrimental. More recently, Daneshazari et al. (2023) reported α‐hemolytic *B. subtilis* isolates that fulfilled the required safety criteria for probiotic evaluation. Taken together, the current literature and the absence of β‐hemolytic isolates in the present study support the selection of α‐hemolytic and γ‐hemolytic isolates for further probiotic characterization.

#### 3.3.4 Determination of Virulence Genes

Members of the *B. cereus* group, which belong to the genus *Bacillus*, are recognized as pathogens and are associated with two major types of foodborne illness: the emetic and diarrheal syndromes. The emetic toxin cereulide is a heat‐stable, ring‐structured peptide synthesized in contaminated foods by a non‐ribosomal peptide synthetase encoded by the *ces* gene. In contrast, diarrheal symptoms result from a variety of enterotoxins produced in the intestine after ingestion of the bacteria, including hemolysin BL (HBL), non‐hemolytic enterotoxin (NHE), cytotoxin K (CytK), enterotoxin T (BceT), and enterotoxin FM (EntFM) (Bianco et al. 2023; Kyei‐Poku et al. 2007). The non‐hemolytic enterotoxin (NHE) is a three‐component enterotoxin encoded by the *nheA*, *nheB*, and *nheC* genes. The presence of all three components is required for biological activity (Cai et al. 2017). Similarly, HBL is a three‐component enterotoxin consisting of two lytic proteins, L1 and L2 (encoded by *hblD* and *hblC*), and a binding protein B (encoded by *hblA*). As with NHE, the presence of all three components is required for biological activity (Reis et al. 2013). CytK, EntFM, and BceT are single‐component toxins associated with the diarrheal syndrome. CytK is a potent cytotoxic and hemolytic toxin capable of forming pores in the lipid bilayer of human intestinal epithelial cells, leading to necrotic effects (Castiaux et al. 2015). Although BceT, encoded by the *bceT* gene, has been classified as a diarrheal enterotoxin, its contribution to food poisoning remains unclear. Several studies have reported that the BceT protein lacks measurable biological activity and does not contribute to toxicity (Gdoura‐Ben Amor et al. 2019).

All of the above‐mentioned virulence genes were screened in the genomic DNA of the 53 isolates, and none of the isolates carried any of the target virulence genes (Table 3). Representative agarose gel electrophoresis images of the PCR assays are provided in Figure S1. The absence of these virulence genes, together with the phenotypic safety assessment, further supports the suitability of the isolates for food‐related applications.

#### 3.3.5 Antibiotic Susceptibility

The inhibition zone diameters and susceptibility classifications of all isolates, together with the commercial probiotic strains, are presented in Table 4. According to the current EUCAST criteria, all isolates were categorized as “susceptible, increased exposure (I)” to both ciprofloxacin and levofloxacin. In addition, only isolate #38 exhibited resistance to erythromycin, whereas all remaining isolates were susceptible to the other antibiotics tested. The commercial probiotic strains CL, CO, and BCE displayed susceptibility profiles comparable to those of the isolates. Overall, the antibiotic susceptibility profiles indicated susceptibility to the tested antibiotics for all isolates, except for erythromycin resistance observed in isolate #38. Resistance to erythromycin by a *Bacillus licheniformis* strain was also reported (Golnari et al. 2024). The findings in the current study support the safety assessment of the selected isolates for probiotic applications, while the ciprofloxacin and levofloxacin results should be interpreted according to the current EUCAST definition of susceptible, increased exposure (I). According to the current EUCAST definitions, the “I” category corresponds to “susceptible, increased exposure” rather than acquired resistance (EUCAST, 2025; Kahlmeter et al. 2019). Therefore, the classification of all isolates as “I” for ciprofloxacin and levofloxacin should be interpreted within this context. Since the present study was based on phenotypic susceptibility testing, the underlying mechanisms responsible for this classification were not investigated. Further molecular or genomic studies would be required to clarify the basis of this phenotype.

**TABLE 4:** Antibiotic susceptibility of the isolates determined according to EUCAST Breakpoint tables Version 15.0.

Antibiotics	EUCAST v.15 zone diameter breakpoints (mm)		Antibiotic sensitivity of the isolates (mm)	
	≥ *S*	< *R*	Minimum	Maximum
Ciprofloxacin (CIP‐5)*	50	23	30.5 ± 3.5	39.0 ± 0.5
Clindamycin DA‐2	17	17	22.0 ± 0.9	36.5 ± 2.1
Erythromycin E15	24	24	18.5 ± 0.7	35.0 ± 1.4
İmipenem IPM‐10	30	30	44.0 ± 0.0	51.0 ± 1.4
Levofloxacin LEV‐5*	50	23	30.0 ± 0.0	36.5 ± 3.5
Linezolid LNZ‐10	22	22	28.0 ± 4.1	36.0 ± 0.0
Meropenem MEM‐10	25	25	29.5 ± 0.7	43.5 ± 2.1
Vancomycin VA‐5	10	10	19.0 ± 1.4	25.5 ± 3.5

* Isolates categorized as screen negative can be reported “susceptible, increased exposure” (I) to ciprofloxacin and levofloxacin. Data are presented as mean ± standard deviation (SD) from three independent experiments (*n* = 3).

### 3.4 Probiotic Properties

#### 3.4.1 Bile Salt Tolerance

Bile salt tolerance is one of the primary criteria for the selection of probiotic strains. Probiotics are naturally exposed to bile salts in the intestine; therefore, their survival at the highest possible level under such conditions is critically important. Bile tolerance assays are commonly performed using 0.3% bile salt, as this concentration closely mimics the physiological bile conditions in the human small intestine. Therefore, 0.3% bile is widely accepted as a critical threshold concentration for evaluating the bile resistance of potential probiotic microorganisms (Dabiré et al. 2022).

**FIGURE 1:**
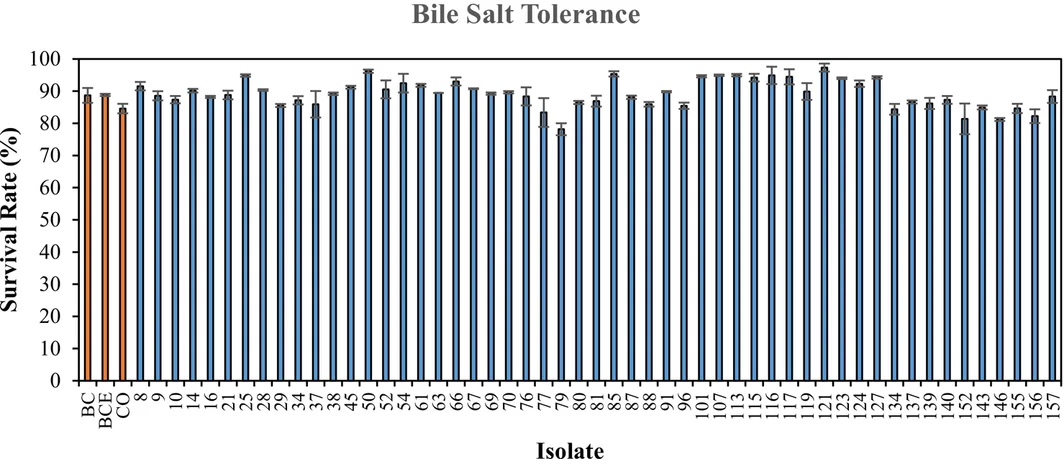
Survival rates of *Bacillus* isolates in the presence of 0.3% bile salts after 3 h of incubation at 37°C. Orange bars represent commercial probiotic control strains (BC, BCE, and CO). Data are presented as mean ± standard deviation (SD) from three independent experiments (*n* = 3). Error bars represent SD.

As shown in Figure 1, the isolates exhibited remarkably high survival rates in the presence of 0.3% bile salts. Among them, isolate #79 displayed the lowest tolerance with a survival rate of 78.2% ± 1.9%, whereas isolate #121 showed the highest resistance at 97.3%. The commercial *Bacillus* probiotics BEC, BCE, and CO used as reference strains (orange bars in Figure 1) had survival rates of 88.7% ± 2.3%, 88.8% ± 0.3%, and 84.6% ± 1.5%, respectively. The mean bile tolerance was 89.14% ± 4.24%, demonstrating the overall high bile salt tolerance of the isolates. Many of the isolates demonstrated bile tolerance levels comparable to or exceeding those of the commercial probiotic strains. Collectively, these results strongly support the probiotic potential of all 53 isolates tested, emphasizing their promising functional capability to withstand intestinal physiological conditions.

High bile salt resistance observed in microorganisms has been associated with their double‐layered cell membrane structure, which enables enhanced tolerance to harsh environmental conditions. A survival rate of 75% after 2 h of exposure to 0.3% bile has been proposed as the minimum threshold for defining acceptable bile tolerance in potential probiotic strains (Suwannaphan 2021). In a previous study, *B. cereus* F20 and *Bacillus tequilensis* F44 isolated from a traditional fermented African food (soumbalo) were reported to maintain cell viability at rates of 87.91% and 52.69%, respectively, after 3 h of exposure to 0.3% bile salts (Dabiré et al. 2022). In the present study, all isolates exceeded this critical threshold, demonstrating strong probiotic potential.

#### 3.4.2 Simulated Gastric Conditions

**FIGURE 2:**
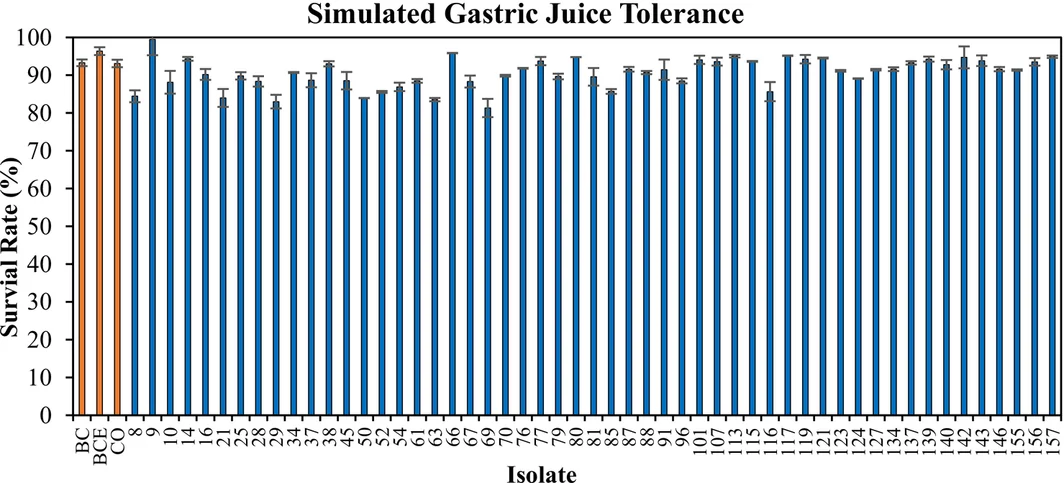
Tolerance of *Bacillus* isolates to simulated gastric juice (0.3% pepsin, pH 2.5) after 3 h of incubation at 37°C. Orange bars indicate commercial probiotic control strains (BC, BCE, and CO). Data are presented as mean ± standard deviation (SD) from three independent experiments (*n* = 3). Error bars represent SD.

Survival under simulated gastric conditions is an essential functional property of probiotic candidates, as gastric acidity represents a major barrier during gastrointestinal transit (Daneshazari et al. 2023). Similar to the bile salt resistance test, all isolates exhibited remarkably high resistance to simulated gastric juice. The lowest survival rate was observed in isolate #69, with 81.3% ± 2.4% viable cells, whereas the highest survival rate was recorded for isolate #9, reaching 99.5% ± 4.2% (Figure 2). The commercial reference strains BC, BCE, and CO showed survival rates of 93.3% ± 0.9%, 96.4% ± 1.0%, and 93.1% ± 1.0%, respectively. Across all isolates, the mean survival rate under simulated gastric conditions was 90.95% ± 3.88%. In general, all isolates demonstrated strong tolerance to simulated gastric conditions, a critical functional attribute for probiotic candidates highlighting their robust potential to survive the harsh environment of the gastrointestinal tract and supporting their suitability for further probiotic characterization. The results obtained in this study are comparable to those reported by Dabiré et al. (2022) and Daneshazari et al. (2023).

#### 3.4.3 Cell Surface Hydrophobicity

**FIGURE 3:**
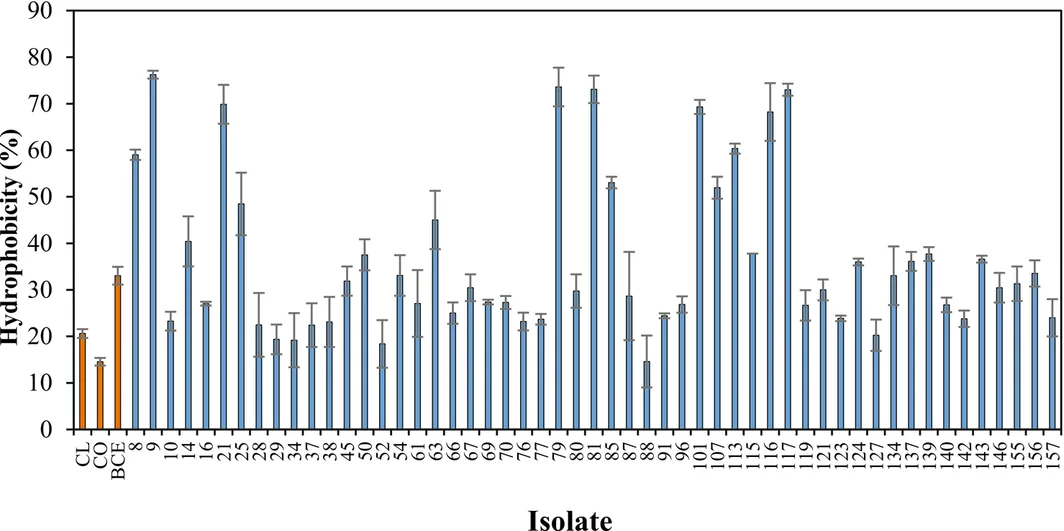
Cell surface hydrophobicity values of *Bacillus* isolates were determined using the Bacterial Adhesion to Hydrocarbons (BATH) method with n‐hexadecane. The dashed reference line indicates the 50% hydrophobicity threshold associated with strong adhesion capacity. Hydrophobicity of commercial probiotic strains (CL, CO, and BCE) is also shown for comparison. Data are presented as mean ± standard deviation (SD) from three independent experiments (*n* = 3). Error bars represent SD.

The hydrophobicity assay, also referred to as the Bacterial Adhesion to Hydrocarbons (BATH) test, is an important criterion for evaluating the probiotic potential of microorganisms. Hydrophobicity and auto‐aggregation are closely associated with a strain's ability to adhere to intestinal epithelial cells, which is considered a critical functional property of probiotics. The adherence of probiotic microorganisms to the gut epithelial surface is directly influenced by their cell surface hydrophobicity and auto‐aggregation capacity (Ruiz Sella et al. 2021). Therefore, probiotic bacterial strains are expected to exhibit high levels of hydrophobicity. As shown in Figure 3, the hydrophobicity levels of the isolates determined using n‐hexadecane varied considerably among strains. The lowest hydrophobicity was observed in isolate #88 (14.6% ± 5.6%), while isolate #9 exhibited the highest value (76.2% ± 0.8%). A total of 11 isolates (#8, 9, 21, 79, 81, 85, 101, 107, 113, 116, and 117) displayed hydrophobicity values greater than 50%, which indicates strong cell surface hydrophobicity. In contrast, the commercial probiotic strains used as controls (CL, CO, and BCE) showed markedly lower hydrophobicity levels, recorded as 20.6% ± 1.0%, 14.6% ± 0.8%, and 33.0% ± 1.9%, respectively. When compared with these commercial strains, the *Bacillus* isolates demonstrated substantially higher hydrophobicity, highlighting their stronger potential for adhesion to intestinal epithelial surfaces and thus strengthening their relevance as promising probiotic candidates. The mean hydrophobicity value was 35.75% ± 16.94%.

Large variations in hydrophobicity values have been reported to arise not only from strain‐specific differences in the expression of cell surface proteins but also from environmental factors that can influence their expression. In a study, the hydrophobicity levels of three probiotic LAB (Lp9 isolate, *Lactobacillus johnsonii* LA1, and *Lactobacillus acidophilus* LA7) were determined as 37.7%, 46.8%, and 56.7%, respectively, using n‐hexadecane (Kaushik et al. 2009). Tulumoğlu et al. (2023) calculated hydrophobicity levels ranged from 24% to 56% for 15 LAB isolated from goat milk. Additionally, hydrophobicity values of 65.9% and 25.6% were reported for *L. acidophilus* B14 and *L. casei* B22 isolated from Iranian traditional cheese (Barzegar et al. 2021). *Lactobacillus fermentum* exhibited a hydrophobicity value of 11.69% when tested with n‐hexadecane (Çeti̇nkaya et al. 2020). Daneshazari et al. (2023) determined the hydrophobicity of two *B. subtilis* strains using chloroform, toluene, and ethyl acetate, and they reported the hydrophobicity values between 42.44% and 60.93%. When the current findings were compared with the literature data as well as with the commercial *Bacillus* strains used as controls, the hydrophobicity results of the isolates investigated in this study were found to be comparable or even superior in most cases. These results further reinforce the strong adhesion potential of the isolates and highlight their suitability as promising probiotic candidates.

#### 3.4.4 Auto‐Aggregation

**FIGURE 4:**
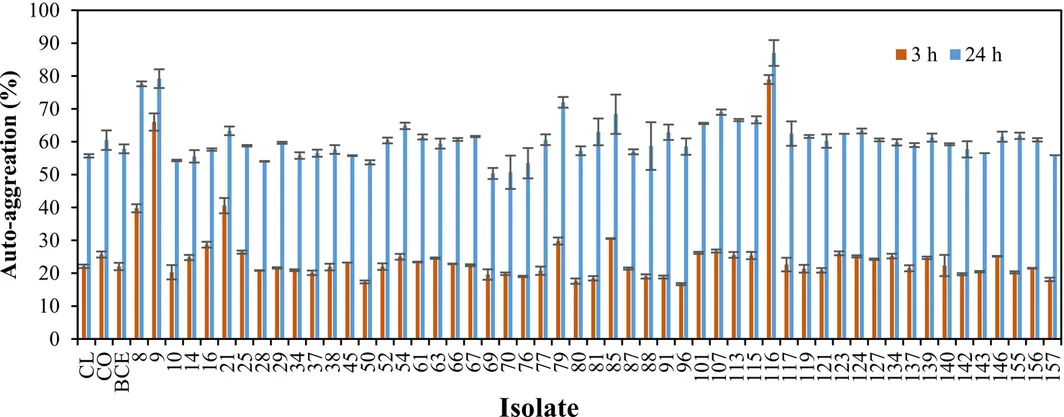
Auto‐aggregation ability of *Bacillus* isolates measured after 3 and 24 h of incubation at 37°C. The auto‐aggregation percentage was calculated from the decrease in absorbance of the upper cell suspension at 600 nm. A substantial increase in auto‐aggregation was observed over time, indicating enhanced cell–cell interaction and potential for intestinal colonization. Commercial probiotic strains (CL, CO, and BCE) are included for comparison. Data are presented as mean ± standard deviation (SD) from three independent experiments (*n* = 3). Error bars represent SD.

The auto‐aggregation of probiotic bacteria provides insight into their ability to colonize the intestinal tract and compete with intestinal pathogens (Jeon et al. 2018). Strains with higher auto‐aggregation capacity are more likely to proliferate efficiently, and increased cell density further enhances adhesion to host epithelial cells (Ruiz Sella et al. 2021). In this study, the auto‐aggregation abilities of all 53 isolates were evaluated after 3 h and 24 h incubation periods (Figure 4). After 3 h, the auto‐aggregation values ranged between 16.7% ± 0.3% (isolate #96) and 78.9% ± 1.3% (isolate #116). A remarkable increase was observed after 24 h, with minimum and maximum values reaching 50.4% ± 1.7% (isolate #69) and 87.0% ± 3.9% (isolate #116), respectively. Notably, isolate #116 consistently exhibited the highest auto‐aggregation values at both incubation times, indicating a particularly strong auto‐aggregation capacity among the tested isolates. The commercial probiotic strains CL, CO, and BCE exhibited auto‐aggregation values of 22.1% ± 0.5%, 25.7% ± 0.9%, and 22.0% ± 1.1% at 3 h, and 55.7% ± 0.5%, 60.5% ± 3.0%, and 57.8% ± 1.3% at 24 h. The mean auto‐aggregation value at 24 h was 61.03% ± 6.5%. These results clearly indicate that the auto‐aggregation performance of the commercial strains was comparable to that of the *Bacillus* isolates obtained in this study. Thus, the isolates demonstrated strong cell–cell interaction, supporting their potential for intestinal colonization and for competitive exclusion of pathogens.

Previous studies have reported considerable variation in the auto‐aggregation capacities of *Bacillus* strains. Konuray Altun and Erginkaya (2021) reported 24 h auto‐aggregation values of 62.19%, 25.64%, and 17.84% for *B. coagulans* isolates. Similarly, Daneshazari et al. (2023) observed lower auto‐aggregation values after 4 h (39.10% and 48.78%), which increased to approximately 62% after 24 h. Borthakur et al. (2025) reported relatively low 3 h auto‐aggregation values, ranging from 10.74% to 15.49% among five newly isolated *Bacillus* strains. On the other hand, Lei et al. (2024) reported high auto‐aggregation capacities (70.2%–84.46%) after 25 h for three *Bacillus pumilus* strains. When compared with the reported literature values and the commercial *Bacillus* strains used as controls in this study, the isolates examined here exhibited strong auto‐aggregation capability. These findings confirm that the isolates possess an essential trait associated with probiotic functionality, further supporting their potential to effectively colonize the gastrointestinal tract and competitively exclude pathogenic microorganisms.

#### 3.4.5 Biofilm Formation

**FIGURE 5:**
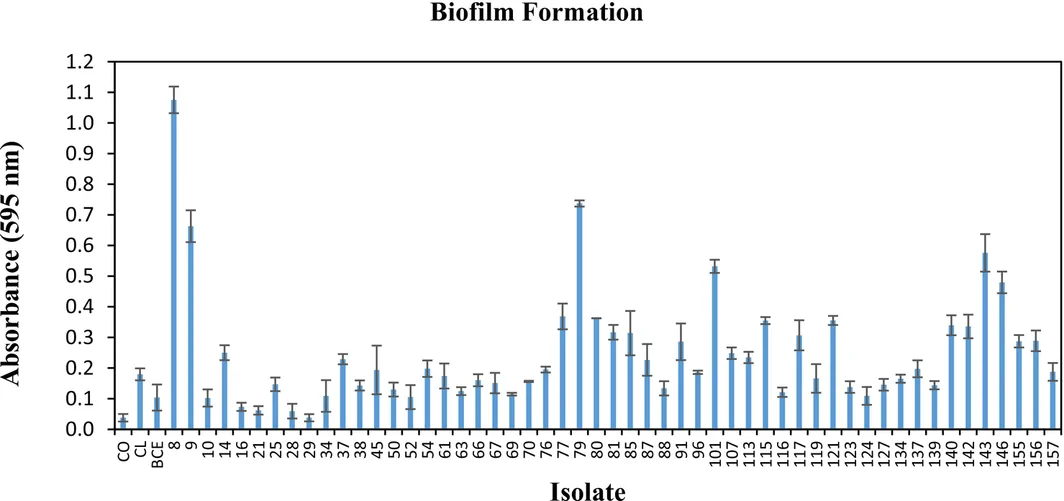
Biofilm formation capacity of *Bacillus* isolates was assessed using the crystal violet microtiter plate assay. Absorbance values measured at 595 nm were used to classify biofilm formation as weak (abs < 0.124), moderate (abs: 0.124–0.240), or strong (abs > 0.240). Commercial probiotic control strains (CO, CL, and BCE) exhibited weak‐to‐moderate levels of biofilm formation. Data represent biofilm‐forming ability after incubation at 37°C for 24–72 h. Data are presented as mean ± standard deviation (SD) from three independent experiments (*n* = 3). Error bars represent SD.

The ability of probiotic microorganisms to survive and colonize the intestinal tract is also closely associated with their biofilm‐forming capacity. Through biofilm formation, cells create a protective barrier that helps them withstand harsh gastrointestinal conditions. The extracellular matrix of biofilms is primarily composed of polysaccharides, along with other macromolecules such as proteins, lipids, and nucleic acids. In addition to facilitating attachment to the intestinal epithelium, biofilms prevent enteropathogens from adhering to host tissues (Ruiz Sella et al. 2021). Biofilm formation by the isolates is shown in Figure 5. Accordingly, 10 isolates were categorized as weak biofilm formers, 23 isolates as moderate, and 20 isolates as strong biofilm formers. The commercial strains CO, CL, and BCE exhibited weak or moderate levels of biofilm formation. These results clearly demonstrate that the majority of the isolates possessed a superior biofilm formation ability compared to the commercial probiotic strains. The mean absorbance value was 0.29 ± 0.2. In a study by Konuray Altun and Erginkaya (2021), *B. coagulans* isolates obtained from various fruits showed absorbance values ranging from 0.13 to 0.23 for biofilm formation, corresponding to weak‐to‐moderate biofilm‐forming capacity. Camele et al. (2019) investigated the biofilm‐forming ability of *B. mojavensis* in two different media: a mineral‐enriched medium and a casamino acid–supplemented Luria‐Bertani medium. They reported absorbance values of 0.287 after 24 h in the mineral‐enriched medium and 1.081 in the casamino acid‐supplemented medium, indicating a substantial influence of nutritional conditions on biofilm development. Similarly, Dabiré et al. (2022) reported considerable variation in the biofilm‐forming abilities of probiotic *Bacillus* strains, which is consistent with the findings of the present study.

### 3.5 Selection of the Isolates

Based on the preliminary screening of probiotic properties, using a heat map for 53 isolates, 12 isolates (isolates #8, 9, 21, 25, 79, 81, 85, 101, 107, 113, 116, and 117) were selected as probiotic candidates. The main probiotic properties of the selected isolates are listed in Table 5. The selected isolates were obtained from the tarhana samples of 5 different cities: Kocaeli (2), Tokat (2), Maraş (3), Gaziantep (2), and Uşak (3). These isolates with promising collective probiotic properties will be investigated further.

**TABLE 5:** Selection of the isolates with superior probiotic attributes.

Isolate number	Source of the sample (City)	Aggregation (%)	Hydrophobicity (%)	Tolerence to simulated gastric system (%)	Bile salt tolerence (%)	Biofilm formation (abs)	Lecithinase activity	DNase activity	Hemolizis
8	Kocaeli	77.6 ± 0.7	59.0 ± 1.1	84.4 ± 1.6	91.5 ± 1.3	1.08 ± 0.04	—	—	*α*
9	Kocaeli	79.2 ± 2.8	76.2 ± 0.8	99.5 ± 4.2	88.6 ± 1.4	0.66 ± 0.05	—	—	*α*
21	Tokat	63.3 ± 1.3	69.9 ± 4.2	84.0 ± 2.4	88.8 ± 1.4	0.06 ± 0.01	—	—	*α*
25	Tokat	58.8 ± 0.2	48.5 ± 6.7	89.8 ± 1.0	94.9 ± 0.4	0.15 ± 0.02	—	—	*γ*
79	Maraş	72.0 ± 1.6	73.6 ± 4.2	89.7 ± 0.7	78.2 ± 1.9	0.74 ± 0.01	—	—	*γ*
81	Maraş	63.0 ± 4.1	73.1 ± 3.0	89.6 ± 2.3	86.9 ± 1.7	0.32 ± 0.02	—	—	*α*
85	Maraş	68.4 ± 6.0	53.1 ± 1.3	85.7 ± 0.6	95.4 ± 0.8	0.31 ± 0.07	—	—	*α*
101	Gaziantep	65.6 ± 0.2	69.3 ± 1.5	94.1 ± 1.1	94.6 ± 0.3	0.53 ± 0.02	—	—	*α*
107	Gaziantep	69.0 ± 0.8	51.9 ± 2.4	93.6 ± 1.0	94.9 ± 0.2	0.25 ± 0.02	—	—	*γ*
113	Uşak	66.6 ± 0.4	60.3 ± 1.1	95.0 ± 0.3	95.0 ± 0.4	0.23 ± 0.02	—	—	*α*
116	Uşak	87.0 ± 3.9	68.2 ± 6.2	85.6 ± 2.5	94.9 ± 2.7	0.12 ± 0.02	—	—	*γ*
117	Uşak	62.5 ± 3.7	73.0 ± 1.3	95.1 ± 0.1	94.5 ± 2.4	0.31 ± 0.05	—	—	*γ*

*Note:* Data are presented as mean ± standard deviation (SD) from three independent experiments (*n* = 3).

## 4 Conclusion

This study demonstrated that the *Bacillus* isolates from tarhana exhibited multiple key characteristics required for effective probiotic functionality. All isolates showed high survival rates under simulated gastrointestinal conditions, and in the bile salt medium. In addition, strong adhesion‐related traits such as hydrophobicity, auto‐aggregation, and biofilm formation were observed in a substantial number of isolates, in many cases exceeding the performance of commercial probiotic *Bacillus* strains. The combined phenotypic, biochemical, and pathogenicity test results further confirmed that the isolates belonged to spore‐forming *Bacillus* species, which are recognized as safe microorganisms for functional food applications. Overall, the robust gastrointestinal stress tolerance and adhesion abilities demonstrated by the isolates in this study strongly support their potential as promising probiotic candidates for further evaluation and utilization in the food and health‐related industries. Other studies including genomic characterization, other probiotic attributes, and functional activity analysis (e.g., antimicrobial potential, and enzyme production) are in progress.

## Author Contributions

**Ayşe Avcı:** conceptualization, investigation, funding acquisition, project administration, supervision, methodology, writing – original draft, writing – review and editing, formal analysis, data curation. **Esma Alemdar:** investigation, data curation, formal analysis, methodology. **Elif Sezer:** investigation, writing – review and editing, formal analysis, data curation. **İnci Cerit:** investigation, writing – review and editing, formal analysis, data curation. **Fikriye Alev Akçay:** investigation, formal analysis, writing – review and editing, data curation. **İbrahim Çakır:** writing – review and editing, methodology, investigation.

## Funding

This work was supported by the Scientific and Technological Research Council of Türkiye (TÜBİTAK) (Grant Number 123O924).

## Conflicts of Interest

The authors declare no conflicts of interest.

## Supporting information


**Figure S1:** Representative agarose gel images showing the amplification of the virulence‐associated genes: (a) *bceT* gene; (b) *entFM* gene; (c) *hblA* gene; (d) *hblC* gene; (e) *hblD* gene; (f) *nheA* gene. Positive controls: C1, *Bacillus cereus* EKT1; C2, *Bacillus cereus* SBT8; C3, *Bacillus* sp. KET2. For all panels, the first lane represents the DNA ladder, followed by the positive control strains (C1–C3). All subsequent lanes correspond to the tested isolates, whose identification numbers are shown above the respective lanes.

## Data Availability

The data that support the findings of this study are available from the corresponding author upon reasonable request.
