# Supplementary material for: Safety Evaluation of Bacillus Isolates From Fermented Tarhana With Probiotic Potential
Source: Food Sci Nutr. 2026 Sep 24;14(9):e72372. doi: 10.1002/fsn3.72372 (PMC13602818; doi:10.1002/fsn3.72372)
Supplement: Supplementary file 1 [file FSN3-14-e72372-s001.docx]

**Supplementary Figure S1**. Representative agarose gel images showing the amplification of the virulence-associated genes: (a) *bceT* gene; (b) *entFM* gene; (c) *hblA* gene; (d) *hblC* gene; (e) *hblD* gene; (f) *nheA* gene. Positive controls: C1: *Bacillus cereus* EKT1; C2: *Bacillus cereus* SBT8; C3: *Bacillus* sp. KET2. For all panels, the first lane represents the DNA ladder, followed by the positive control strains (C1–C3). All subsequent lanes correspond to the tested isolates, whose identification numbers are shown above the respective lanes.

**(b)**

**(a)**

**(c)**

**(d)**

**(e)**

**(f)**
